# Supplementary material for: Pleiotropic Roles of the Orthologue of the Drosophila melanogaster Intersex Gene in the Brown Planthopper
Source: Genes (Basel). 2021 Mar 7;12(3):379. doi: 10.3390/genes12030379 (PMC8000406; doi:10.3390/genes12030379)
Supplement: Supplementary file 1 [file genes-12-00379-s001.pdf]

genes

Table S1. The main primers used in this study.

Primer name	Sequence (5'-3')*	Purpose	Length (bp)
Nlix^{L}-RNAi-F-1	T7-TTCTGGATTGAACAGTGGCAAT	*Nlix*^{L}-1 dsRNA synthesis	261bp
Nlix^{L} -RNAi-R-1	T7-CAATGTTTCTCTTAGTTGACCG		
Nlix^{L} -RNAi-F-2	T7-AATAATTTGGTGGACGTTGG	*Nlix*^{L}-2 dsRNA synthesis	318bp
Nlix^{L} -RNAi-R-2	T7-AGGGTTCTGAATGTTGTGTG		
Nlix^{L}-F	ATGTTCTGGATTGAACAGTGG	PCR for *Nlix*^{L} ORF	654bp
Nlix^{L} -R	AGTTGGTGGAAAGCAGCTTCA		
Nlix^{S}-F	TTCTGGATTGAACAGTGGCAAT	PCR for *Nlix*^{S} ORF	311bp
Nlix^{S}-R	GTTTTACATTATATTTAATAATGC		
Q*Nlix*^{L} -F-1	GACAACCGAATATGCCAGGACAG	RT-qPCR for *Nlix*^{L}-1	97bp
Q*Nlix*^{L} -R-1	AGTTGACCGACAAGAGATTTGACT		
Q*Nlix*^{L} -F-2	ACTCTCAAAACAGCGGCCA	RT-qPCR for *Nlix*^{L}-2	172bp
Q*Nlix*^{L} -R-2	TTCCTTGGTTCAGGCACTCC		
QNl18S-F	CGCTACTACCGATTGAA	qRT-PCR for 18s	132bp
QNl18S-R	GGAAACCTTGTTACGACTT		

*T7, 5 ´ -TAATACGACTCACTATAGGGAGA-3 ´

Table S2: The 32 IX homologs derived from related species.

Species	GenBank accession number
Trypoxylus dichotomus	BBE20904.1
Tribolium castaneum	EFA01132.1
Photinus pyralis	XP_031347699.1
Sitophilus oryzae	XP_030765845.1
Bombyx mori	NP_001037105.1
Papilio polytes	BAM20686.1
Helicoverpa armigera	XP_021191665.1
Bombyx mandarina	XP_028038928.1
Maruca vitrata	ACS12898.1
Drosophila melanogaster	AAN37397.1
Drosophila virilis	AAV65894.1
Culex quinquefasciatus	XP_001849607.1
Megaselia scalaris	AAV65895.1
Anopheles darlingi	ETN59144.1
Oncopeltus fasciatus	AEM16993.1
Bemisia tabaci	QAB02863.1
Halyomorpha halys	XP_014282369.1
Nilaparvata lugens	XP_022205962.1
Cimex lectularius	XP_014249089.1
Melanaphis sacchari	XP_025204986.1
Aphis gossypii	XP_027848160.1
Sipha flava	XP_025417563.1
Apis cerana	XP_028522479.1
Dufourea novaeangliae	KZC13858.1
Bombus impatiens	XP_033180622.1
Megalopta genalis	XP_033334678.1
Osmia lignaria	XP_034175582.1
Coptotermes formosanus	GFG29436.1
Zootermopsis nevadensis	XP_021926959.1
Cryptotermes secundus	XP_023706449.1
Thrips palmi	XP_034248708.1
Frankliniella occidentalis	XP_026279401.1

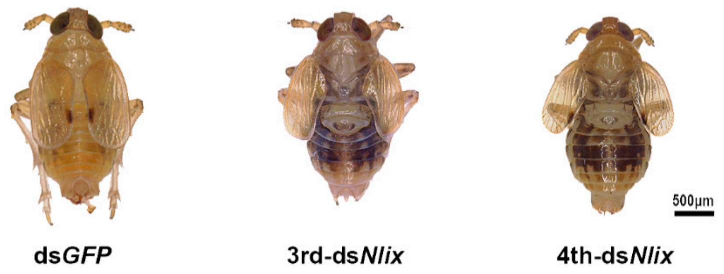

Figure S1. The influence of *Nlix* on BPH wings development. The male individuals developed small and abnormal forewings after injection of ds*Nlix* in 3rd or 4th instars. ds*GFP* was injected as a negative control for the nonspecific effects of dsRNA.
